# Supplementary material for: Site-Specific Radiolabeling of a Human PD-L1 Nanobody via Maleimide–Cysteine Chemistry
Source: Pharmaceuticals (Basel). 2021 Jun 8;14(6):550. doi: 10.3390/ph14060550 (PMC8228271; doi:10.3390/ph14060550)
Supplement: Supplementary file 1 [file pharmaceuticals-14-00550-s001.zip › pharmaceuticals-1222772-supplementary.pdf]

Supplementary Information

1. QCs and Characterization of the NOTA-mal-Nb

SEC

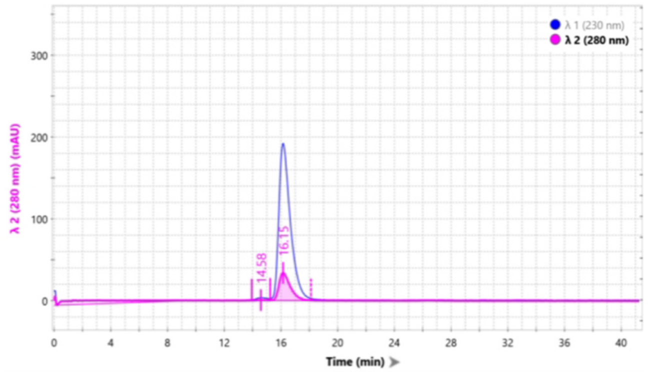

Figure S1. Size exclusion chromatography analysis (UV profile) of the purified NOTA-mal-(hPD-L1) Nb showing a 98% purity at 280 nm (pink line). Analysis at 230 nm (blue line) gives similar results.

SDS PAGE

2 µg and 10 µg of the conjugated Nb (NOTA-mal-hPD-L1) was analysed under non-reducing (2x LaemmLi Sample Buffer, Bio-Rad) and reducing conditions (LaemmLi Sample buffer supplemented with 5%dithiothreitol, DTT, Bio-Rad). A pre-stained ladder (PageRuler™ Prestained Protein Ladder, 10 to 180 kDa, ThermoFischer Scientific) and Bovin Serum Albumin (BSA, Bio-Rad Protein Assay Standard II) were used as reference on the SDS PAGE gel (Novex™ WedgeWell™ 16% Tris-Glycine Gel, ThermoFisher Scientific).

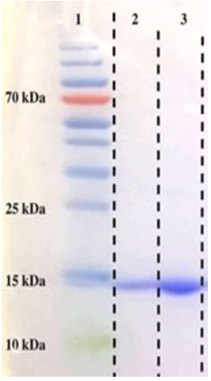

Figure S2. SDS PAGE result. Lane 1 = Pre-stained ladder; Lane 2 = 2 µg of purified NOTA-mal-(hPD-L1) Nb in non-reducing conditions; Lane 3 = 10 µg of NOTA-mal-(hPD-L1) Nb in non-reducing conditions.

2. ESI-Q-ToF

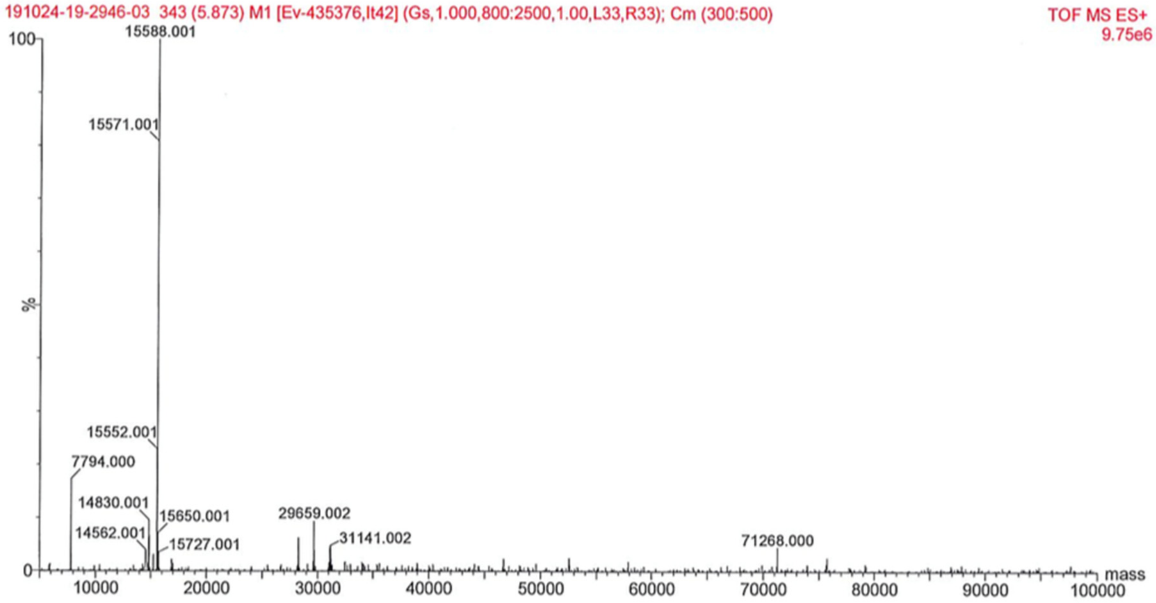

Figure S3. Mass determination analysis of the NOTA-mal-Nb showing the mass peak at 15588 Da (calculated = 15588 Da) and the deamidated compound at 15571 Da. Starting Nb (15165 Da) or Nb-dimer (30330 Da) are not visible

3. Surface Plasmon Resonance

Measurements were performed on a Biacore T200 device (GE Healthcare) at 25°C and using Hepes-buffered saline (HBS; 0.01 M HEPES pH 7.4, 0.15 M NaCl, 3 mM EDTA, 0.005% Tween 20) as running buffer. The recombinant protein was dissolved to 10 µg/mL in 10 mM NaOAc pH 5.0 for immobilization on a CM5 sensor chip using linkage chemistry with 1-(3-(dimethylamino)propyl)-3-ethylcarbodiimide (EDC) and *N*-hydroxy-succinimide (NHS). Unreacted EDC-NHS linkers were blocked with 1 M ethanolamine-HCl.

The modified Nbs were tested for affinity on immobilized human PD-L1 protein in SPR. To this end, 9 different Nb dilutions were allowed to bind to the target protein for 120 sec and dissociation was monitored for 160 sec. The equilibrium dissociation constant K_D was calculated by fitting the obtained sensor-grams to theoretical curves, assuming 1-to-1 binding geometries, using Biacore Evaluation software.

4. Thermostability of the Nb (melting temperature)

The melting temperature of the starting Nb and NOTA-modified Nb was determined using the Protein Melting program of a RealTime PCR machine. Samples were prepared by mixing 12.5 µg of Nb with 7.5 µL of Cypro Orange dye (Thermo fisher, 300 x dilution) in PBS to a 25 µl final volume. Blank samples contained NH4OAc. Samples were prepared in triplicates.

5. Stability Studies

In Vitro Stability Studies of [^{68}Ga]Ga-NOTA-mal-(hPD-L1) Nb

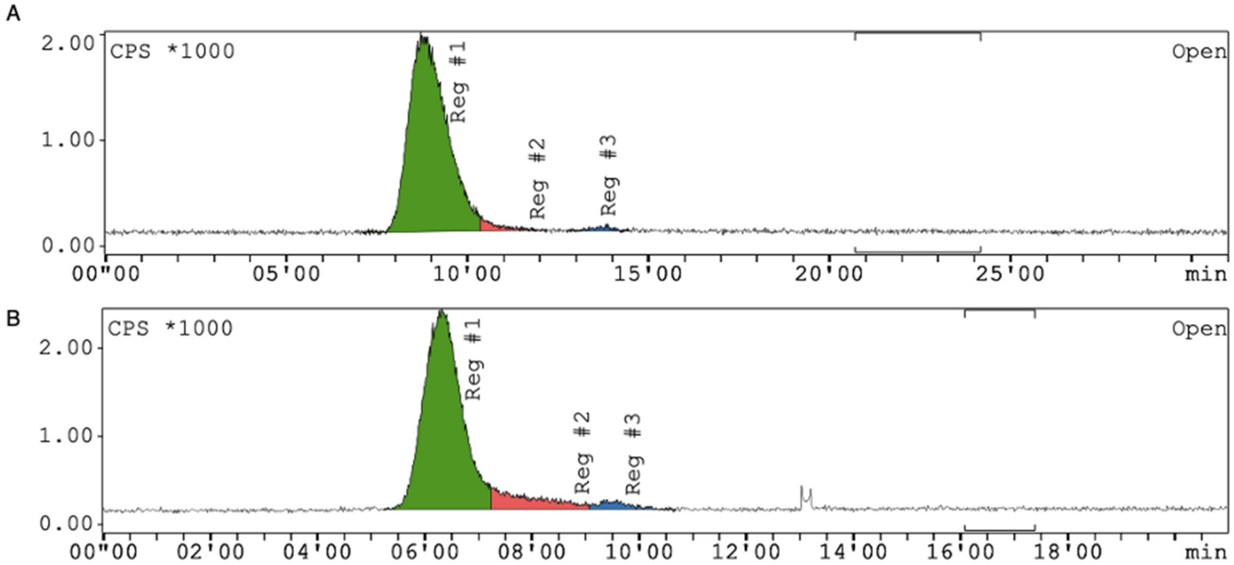

Figure S4. *In vitro* stability study of [^{68}Ga]Ga-NOTA-mal-(hPD-L1) Nb. (**A**) Radio-SEC showing > 95% radiochemical purity of [^{68}Ga]Ga-NOTA-mal-(hPD-L1) Nb after 180 min in injection buffer at room temperature. (**B**) Radio-SEC showing > 85% radiochemical purity of [^{68}Ga]Ga-NOTA-mal-(hPD-L1) Nb after 180 min in human serum at 37°C.

^{68}Ga-labeling and Stability of NOTA-mal-(hPD-L1) Nb after Two Months Storage

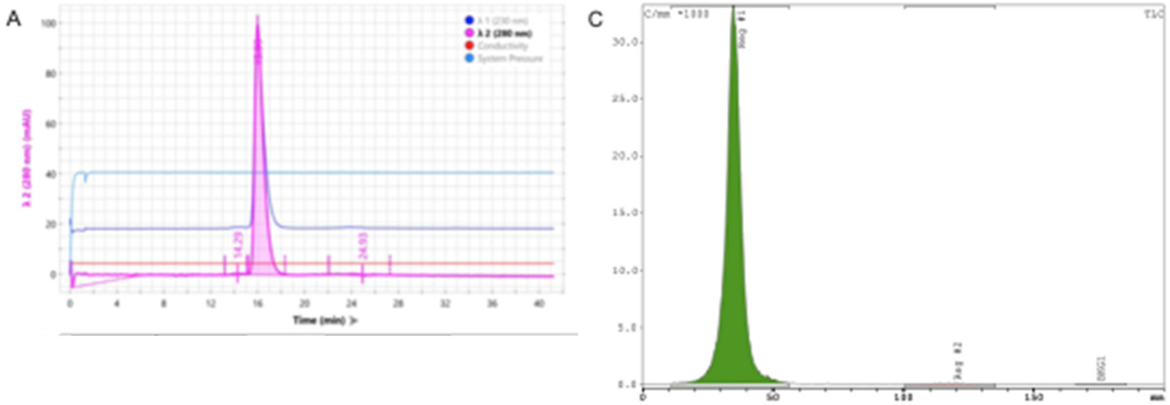

Figure S5. Stability of NOTA-mal-(hPD-L1) Nb after two months storage at -30°C in 0.1 M NH_4OAc. (**A**) Size exclusion chromatography analysis of NOTA-mal-(hPD-L1) Nb at 280 nm showing > 98% purity. Rt(Nb) = 15.99 min, Rt(dimer) = 14.29 min, Rt(degradation) = 21—28 min. (**B**) Radio-iTLC of [^{68}Ga]Ga-NOTA-mal-(hPD-L1) Nb (after purification) after 180 min in injection buffer showing a RCP > 99%. Rf(^{68}Ga-Nb) = 0, Rf(free ^{68}Ga) = 1.

6. Tumor Targeting

Biodistribution Profile

Table S1. Biodistribution and tumor uptake 1 h 20 post-injection of the site-specifically [^{68}Ga]Ga-NOTA-mal-(hPD-L1) Nb in hPD-L1^{POS} and hPD-L1^{NEG} tumor bearing athymic nude mice (N = 12 and N = 6 / group, respectively), mean of %IA/g for each organ or tissue, with the standard deviation (SD).

	hPD-L1^{POS}		hPD-L1^{NEG}	
	%IA/g	SD	%IA/g	SD
Blood	0.38	0.13	0.42	0.03
Heart	0.17	0.12	0.15	0.01
Lungs	0.34	0.13	0.44	0.13
Liver	0.97	0.45	0.87	0.26
Spleen	0.54	0.35	0.36	0.11
Pancreas	0.10	0.03	0.12	0.01
Kidneys	27.90	5.07	23.90	7.63
Stomach (without content)	0.11	0.04	0.13	0.04
Small intestine (without content)	0.24	0.42	0.13	0.03
Large intestine (without content)	0.12	0.03	0.14	0.02
White fat from pelvis	0.17	0.29	0.10	0.03
Muscle	0.08	0.04	0.11	0.04
Bone	0.14	0.06	0.18	0.03
Lymph nodes	0.19	0.06	0.28	0.10
Brown fat	0.13	0.04	0.13	0.03
Tumour	1.86	0.67	0.42	0.03

Flow Cytometry

hPD-L1 expression on the cells from the dissected tumors was assessed. The dissected tumors stored in PBS (max. 12 h) were cut, placed in 5 mL RPMI medium and treated using a gentleMACS™ dissociator. 150 µL of Collagenase from *Clostridium histolyticum* (Sigma Aldrich, 10.000 U/mL in PBS) and 150 µL of Dispase (Sigma Aldrich, 32 mg/mL in water) were added to the mixture and incubated at 37°C for 40 min. 2 µL of DNAse (1 mg/mL in PBS) was added to the mixture and treated 2 times on the gentleMACS™ dissociator. After filtration and centrifugation, red blood cell lysis buffer was added. The mixture was centrifuged, and the pellet was incubated with 100 µL of anti-mouse CD16/32 Antibody (clone 93, BioLegends, 1/200 dilution in PBS/BSA) for 10 min at RT. The pellets were incubated 30 min at 4°C with either 20 µL of isotype control solution (PE-CF594 Mouse IgG1, k Isotype Control, Clone X40 RUO, BD Horizon, 1.6/100 µL of PBS/BSA) or 20 µL of staining solution (PE-CF594 Mouse Anti-Human CD274, Clone MIH1 RUO, BD Horizon, 1.6/100 µL of PBS/BSA). Samples were resuspended in PBS/BSA for FC reading (BD FACSCelesta™, BD Biosciences). % of cells expressing hPD-L1 is measured as the difference between the % of positive cells from the stained sample and the % of positive cells from the isotype control sample.

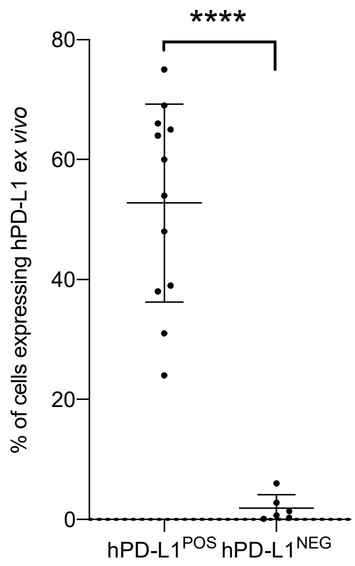

Figure S6. *Ex vivo* assessment of hPD-L1 expression by flow cytometry. hPD-L1 expression in the hPD-L1^{POS} tumors after dissection of the animals, expressed in % of cells expressing hPD-L1, as compared with the hPD-L1^{NEG} tumors (****; $p < 0.0001$).
